# A new, rapid and reproducible method to obtain high quality endothelium in vitro

**DOI:** 10.1007/s10616-012-9459-9

**Published:** 2012-05-10

**Authors:** Nuria Jiménez, Vincent J. D. Krouwer, Jan A. Post

**Affiliations:** Department of Biomolecular Imaging, Institute of Biomembranes, Utrecht University, Padualaan 8, 3584 CH Utrecht, The Netherlands

**Keywords:** Cobblestone, Electron microscopy, Endothelium, HUVEC, Immuno-fluorescence, LDL-uptake, Umbilical vein

## Abstract

Human umbilical vein endothelial cells (HUVECs) cultured in vitro are a commonly used experimental system. When properly differentiated they acquire the so-called cobblestone phenotype; thereby mimicking an endothelium in vivo that can be used to shed light on multiple endothelial-related processes. In the present paper we report a simple, flexible, fast and reproducible method for an efficient isolation of viable HUVECs. The isolation is performed by sequential short trypsinization steps at room temperature. As umbilical cords are often damaged during labor, it is noteworthy that this new method can be applied even to short pieces of cord with success. In addition, we describe how to culture HUVECs as valid cobblestone cells in vitro on different types of extracellular matrix (basement membrane matrix, fibronectin and gelatin). We also show how to recognize mature cobblestone HUVECs by ordinary phase contrast microscopy. Our HUVEC model is validated as a system that retains important features inherent to the human umbilical vein endothelium in vivo. Phase contrast microscopy, immuno-fluorescence and electron microscopy reveal a tight cobblestone monolayer. Therein cells show Weibel-Palade bodies, caveolae and junctional complexes (comparable to the in vivo situation, as also shown in this study) and can internalize human low density lipoprotein. Isolation and culture of HUVECs as reported in this paper will result in an endothelium-mimicking experimental model convenient for multiple research goals.

**Electronic supplementary material:**

The online version of this article (doi:10.1007/s10616-012-9459-9) contains supplementary material, which is available to authorized users.

## Introduction

The endothelium forms a continuous cell monolayer that lines the lumen of blood vessels. By their location and functionality, vascular endothelial cells play a critical role as selective barrier for the transit of water, solutes and cells between blood and underlying tissues. In addition, endothelial cells fulfill other pivotal functions as they are involved in the regulation of the vascular tone and hemostasis and they participate in the inflammatory and immune response. Alterations of the endothelial integrity and functionality may lead to disorders related to atherosclerosis, thrombosis and inflammation (Simionescu and Antohe 2006). The successful culture of endothelial cells started 4 decades ago, and marked the beginning of the modern vascular biology (Nachman and Jaffe 2004). Human endothelial cells cultured in vitro are a simple experimental system that allows for the study of diverse facets of the normal endothelial biology and mechanisms underlying the vascular pathology (Striker et al. 1980). Besides for fundamental research, human umbilical vein endothelial cells (HUVECs) are often the model system of choice for the bio-pharmaceutical industry and preclinical assays since they have several advantages. HUVECs are primary, non-immortalized cells of human origin, they are relatively easy to isolate without contamination of other cell types and umbilical veins are readily available (Manconi et al. 2000).

After the post-natal resection of the umbilical cord, the umbilical vein can be easily cannulated and the endothelium can be detached by enzymatic activity. This approach was first used by Maruyama (Maruyama 1963). However, it was in the 1970′s when it was applied by Jaffe and others to successfully isolate cells that could be propagated in vitro and identified as *bona fide* HUVECs (Gimbrone et al. 1974; Jaffe et al. 1973a, b, 1974). In the last years, several protocols to isolate this cell type have been published (Baudin et al. 2007; Bazzoni et al. 2002; Davis et al. 2007; Laurens and van Hinsbergh 2004; Marin et al. 2001). In all the referred works relatively long pieces of umbilical cord were used to isolate HUVECs, always by collagenase activity at 37 °C. This practice bears some disadvantages; namely (1) pieces of umbilical cord available for cell isolation might be short; (2) incubations of cords in water bath happen under “semi-sterile” conditions; and (3) collagenase might carry enzymatic contaminants and every batch needs to be tested to find out the proper incubation conditions (time, concentration).

Isolated HUVECs can be propagated using different types of endothelial-specific media, supplements and coatings for the culture vessels (Albelda et al. 1989; Chazov et al. 1981; Clark et al. 1986; Gimbrone et al. 1974; Jaffe et al. 1973b; Lewis et al. 1973; Maciag et al. 1981). Confluent HUVECs can differentiate into a monolayer of tightly packed cells, the so-called cobblestone phenotype, that resembles endothelium morphology in vivo (Smeets et al. 1992). Coatings as fibronectin and interstitial collagens I and III favor endothelial cell migration and proliferation (Grant et al. 1990) while basal lamina components, as laminin and collagen IV, promote endothelial cell attachment and differentiation (Grant et al. 1990). Hereby, it seems that a coating resembling the basal lamina could be a good starting point to set up a cobblestone monolayer similar to an endothelium in vivo (Martins-Green et al. 2008).

In this paper we report an optimized, integral protocol to isolate HUVECs in a simple and fast way and to grow them to a versatile cell monolayer that shows strong similarities with the umbilical vein endothelium. The isolation method is less demanding than other previously published ones since it uses ordinary trypsin and manipulation is performed at room temperature (RT). Furthermore, a piece of umbilical cord as short as 10 cm can be used to obtain an appropriate cobblestone culture. Cells can be easily propagated and suitably differentiated, as demonstrated by phase contrast, immuno-fluorescence and electron microscopy. By thorough, qualitative, observation of cells we were able to empirically find simple rules to seed and propagate HUVECs with potential to acquire a cobblestone phenotype in vitro. This can be achieved on surfaces coated with basement membrane matrix, fibronectin or gelatin, following a simple coating procedure by adsorption. The method that we present is a flexible, convenient and reproducible approach to engineer endothelium-mimicking cultures for diverse experimental purposes.

## Materials and methods

### HUVECs isolation by sequential short trypsinizations and establishment of primary cultures

Human umbilical cords (n = 10; from healthy individuals, at term) were obtained from the Department of Obstetrics and Gynecology, Diakonessen Hospital, Utrecht (The Netherlands), with the informed consent of the parents. Our modified isolation protocol was based in the pioneering approaches of Maruyama and Jaffe (Jaffe et al. 1973b; Maruyama 1963). Umbilical cords were collected in Hanks’ balanced salt solution (HBSS; PAA) supplemented with 100 U/ml of penicillin and 100 μg/ml of streptomycin (Invitrogen) and kept at 4 °C until processing. Some current protocols (including Jaffe’s) (Baudin et al. 2007; Gimbrone et al. 1974; Jaffe et al. 1973b) used fresh umbilical cords. In our present study cell isolation was carried out typically 24 h after birth; however, comparable results can be obtained when cells are isolated 48 h after birth (data not shown). This timespan confers more flexibility to the method, as the researchers can plan isolation in a well-suited moment. Cell isolation was performed in a laminar flow hood, with the working area covered with a compress, wearing clean gloves and lab coat. All reagents and materials used were sterile. Before beginning, buffers (bottles of 500 ml) and enzymatic solutions (50 ml/tube) were warmed up to 37 °C in a water bath. Just before use bottles and tubes were decontaminated and introduced in the flow cast. In a Petri dish (Ø 145 mm; Greiner-BioOne), cords were washed by immersion and gentle squeezing in warm HBSS without Ca^2+^ and Mg^2+^ (HBSS^−/−^; PAA). A 1-cm long piece of cord at both ends and any damaged area were resected with a scalpel. Upon measuring, the length of the remaining “intact” cord was typically 10–30 cm. The cord was placed in a dry, clean Petri dish and both ends of the umbilical vein were cannulated. The free end of the cannulae had been previously attached to 4-cm long silicon tubes. These tubes serve as “inlet” and “outlet” to the umbilical vein lumen and can be connected to syringes and clamped when necessary. Cannulae were fastened to the cord using cable ties. Note that the inlet cannula/silicon tube had been filled up with HBSS-/- prior to cannulation to avoid injection of air into the vein lumen. Warm HBSS^−/−^ was injected to wash remains of blood out of the lumen. Then we proceeded to detach the endothelial cells by sequential short trypsinization. With the outlet-tube clamped, we injected, via the inlet, enough trypsin–EDTA (1x, final concentration 0.05 % (trypsin)-0.022 % (EDTA); diluted in HBSS^−/−^ from trypsin–EDTA 10x; #L11-003, PAA) to distend the lumen of the vein (typically 4–5 ml/10-cm of cord; note that approx. 3 ml stayed in the cannulae). The inlet-tube was then clamped. During the incubation (2 min; at RT, in laminar flow cast), the cord was gently massaged. Then, the pressure was released (by allowing cell suspension to flow via the outlet into a syringe), cord was slightly squeezed and fresh trypsin–EDTA was injected again for a 2-min long incubation. The process was repeated 5 times in total. After the last trypsinization, the vein was perfused once with HBSS^−/−^ (by applying serial distensions and pressure releases; via inlet and outlet, sequentially) to recover remaining cells. The cell suspension collected after every step was immediately transferred to one (or eventually several) 50-ml tubes and kept at RT. After the last recovery, we mixed cells with fetal calf serum (10 % final concentration; PAA) to neutralize trypsin. In this way, cells were exposed to the enzymatic activity for about 10 min, at RT. The whole procedure, from vein washing to trypsin neutralization, took less than 15 min. Cells were centrifuged at 250×*g*, 5 min. Supernatant was discarded and pellets were carefully re-suspended in warm endothelial growth medium (EBM-2 plus EGM-2 supplements; Lonza) with 100 U/ml of penicillin and 100 μg/ml of streptomycin. All remainders of the umbilical cords were treated as biohazard according to institutional rules.

HUVECs were transferred to vessels coated with a thin layer of non-gelled Matrigel™. Matrigel is a basement membrane matrix enriched in basal lamina components (Kleinman et al. 1982). As control, HUVECs from some isolations (n = 2) were cultured in paralle l on other coatings widely used to grow this cell type: human fibronectin (Baudin et al. 2007; Laurens and van Hinsbergh 2004) or gelatin, a mixture of derivatives of skin collagen (Bazzoni et al. 2002; Marin et al. 2001). Matrigel (BD Biosciences) was diluted in cold culture medium without serum (final concentration 100 μg/ml) following the manufacturer’s guidelines. Fibronectin from human plasma (Sigma) was prepared in cold HBSS^−/−^ to coat with 1 μg/cm^2^. Gelatin solution (2 % in H_2_O, from bovine skin; Sigma) was warmed up to 37 °C and used undiluted. To coat the culture vessels we followed a procedure which did not require any drying step. In all cases coating solution was added to the culture vessels (125 μl/cm^2^) and left for at least 1 h in the cell incubator to coat by adsorption. Just before cell seeding, the solution was removed; no washing steps were required.

Isolated HUVECs were seeded following a “1:1, cord length:culture surface” rule; for example, cells isolated from a 10-cm long cord were transferred onto a 10-cm^2^ culture surface (e.g. one well of a 6-well plate). Presence of traces of blood and small clusters of endothelial cells made cell counting unreliable at that point. Cells were incubated at 37 °C in a 5 % CO_2_ humidified atmosphere. After 4 h, medium was refreshed and once again the next day. Traces of blood and cell debris were washed away by this procedure. HUVECs (passage 0) were left to grow for 2–3 days in primary culture. In this time cells propagated, reaching typically ~80 % confluence (n = 8), or stopped growing (n = 2) (see Results and Discussion). No microbial contamination was found in any culture.

### Establishment and characterization of the 7-days cobblestone HUVECs model

**Fig. 1:**
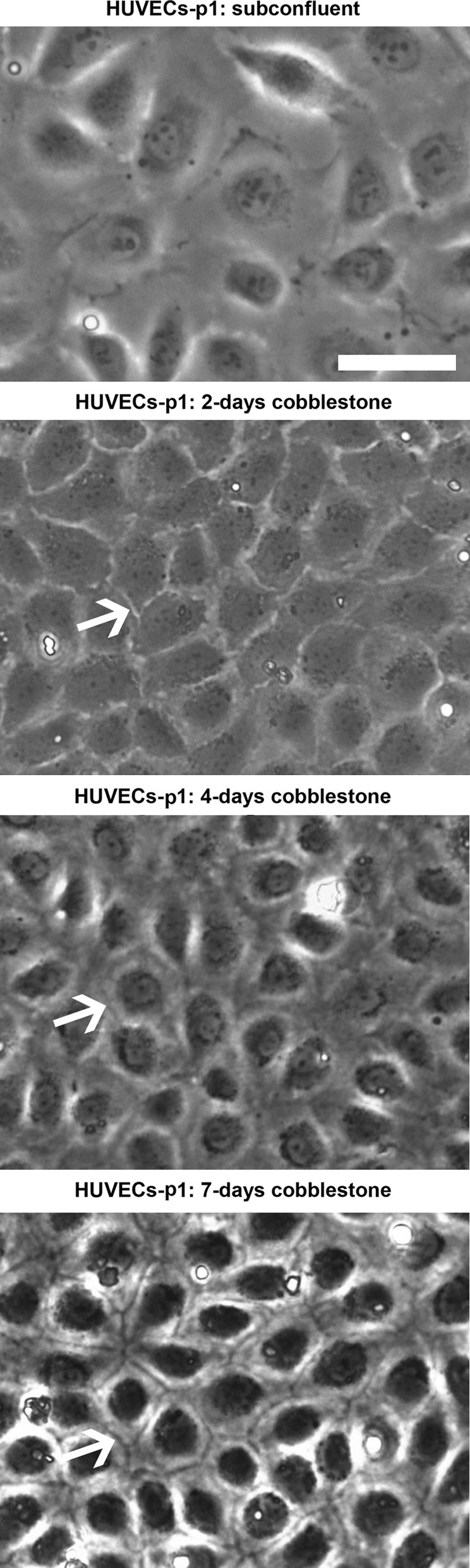
Establishment of a tight, mature cobblestone monolayer assessed by phase contrast microscopy. HUVECs (passage 1, p1) were seeded at 20,000 cells/cm^2^ on ordinary polystyrene culture vessels coated with Matrigel, left to grow and differentiate for several days and regularly monitored by phase-contrast microscopy. At “subconfluent” state cells are spread and divide actively. When the monolayers get confluent, cells are more compact and stop proliferating by contact inhibition. In unripe cobblestone cultures (“2-days cobblestone”) cell limits are bright. As cobblestone cells mature (“4-days cobblestone”), cell limits get *dark* until they become distinct lines (“7-days cobblestone”). *Arrows* *poin*t out cell peripheries. *Scale bar* (applicable to all the *panels*): 50 μm

Viable primary cultures (those reaching ~80 % confluency after 2–3 days in culture; derived from n = 8 cords, see Results and Discussion for further explanation) were used to seed for experiments to obtain cobblestone HUVECs. Cells (passage 0) were trypsinized as follows. Medium was removed, and cells rinsed twice with HBSS^−/−^ at RT. Trypsin–EDTA (0.05–0.022 %, respectively) was added to cover the growth surface and immediately aspirated. Cells were then transferred to the cell incubator at 37 °C. After 1 min, cells were detaching as assessed by phase contrast microscopy. Cells were re-suspended in growth medium (without penicillin/streptomycin) and counted using a hemocytometer. HUVECs (passage 1) were seeded at 20,000 cells/cm^2^ on Matrigel coated culture vessels. Cell growth and cobblestone maturation were regularly monitored with a phase contrast microscope (Leica DMIL) equipped with a CCD camera (Leica EC3) coupled to a computer with the LAS EZ version 1.5.0 software (Leica). After ~4 days cells reached 100 % confluence and started to form a cobblestone layer (see Results and Discussion). About 7 days later, the monolayer showed a tight conformation (Fig. 1). Endothelial growth medium (always without penicillin/streptomycin) was refreshed every 2–3 days and cells were always cultured at 37 °C in a 5 % CO_2_ humidified atmosphere under sterile conditions. The last refreshing was done with medium supplemented with extra serum (10 % fetal bovine serum Gold; PAA), 2 days before cells reached the 7-days cobblestone state.

Characterization of the 7-days cobblestone HUVECs model was done by immuno-fluorescence (n = 2), transmission and scanning electron microscopy (n = 2) as well as by functional assays (LDL uptake; n = 2). For this, cells were cultured on Aclar, a thin transparent copolymer film that can be easily punched, engraved with position marks and used for light, fluorescence and electron microscopy (Jiménez et al. 2010). Aclar can be coated with matrix proteins, allowing for the growth of many cell lines (Jiménez et al. 2006), including HUVECs (Jiménez et al. 2010). Aclar pieces were prepared and attached to 12-well plates exactly as previously reported (Jiménez et al. 2010). Prior to cell seeding, plates were sterilized by ultraviolet light and coated with Matrigel, fibronectin or gelatin as specified in the former section. HUVECs (passage 0) were trypsinized and seeded at 20,000 cells/cm^2^. When monolayers reached the 7-days cobblestone state cells were imaged by phase contrast microscopy and immuno-fluorescent labeled, processed for electron microscopy analysis or used for LDL-uptake assays as explained in the next sections.

### Immuno-fluorescence of HUVECs

HUVECs (7-days cobblestone) were fixed with formaldehyde (from paraformaldehyde; Sigma-Aldrich) at 1 % (wt/vol) in 0.2 M HEPES (Merck) buffer, pH 7.2. The fixative was added 1:1 to the culture medium and kept 5 min at RT before removing. Then fresh fixative was added and left for 20 min at RT. After washing with PBS cells were ready for immuno-labeling. Samples were blocked, quenched and permeabilized in one-step incubation with a cocktail containing 0.5 % BSA, 0.045 % cold water fish gelatin, 50 mM NH_4_Cl and 0.1 % saponin in PBS, for 30 min at RT. The same cocktail was used to dilute primary and secondary antibodies as well as DAPI. Cells were incubated 1 h at RT with 0.5 μg/ml rabbit α-caveolin (#610059; BD Transduction Laboratories), mouse α-claudin-5 at 1:100 (#18-7364; Invitrogen), 2 μg/ml mouse α-VE cadherin/CD144 (#1597; Immunotech) or mouse α-von Willebrand factor at 1:500 ((Pareti et al. 1986); a gift from Prof. P. De Groot, Dept. Hematology, UMCU, Utrecht, The Netherlands). After washing with PBS, cells were incubated 1 h at RT with secondary antibodies conjugated with Alexa Fluor 555 or Alexa Fluor 488 (Molecular Probes) according to the manufacturer’s guidelines. Negative controls were carried out omitting the primary antibody as previously published (Jiménez and Post 2012). HUVECs were washed with PBS before incubation with 2 μg/ml DAPI (Roche Diagnostics) in PBS, 5 min at RT. The samples were washed with PBS and distilled H_2_O and finally mounted with Prolong Gold (Molecular Probes). To this end, Aclar pieces were sandwiched between a glass slide and a coverslip with cells facing the coverslip. Samples were left to cure before analysis. Cells were imaged using a wide-field fluorescence microscope (Provis AX70; Olympus) equipped with a Nikon DXM1200 digital camera (Nikon Instruments Europe). Pictures were captured using the Nikon ATC-1 software (v. 2.63).

### Human LDL purification and labeling with Oregon Green (OG)

Human LDL was isolated from plasma (Bloedbank Midden Nederland) by density-gradient ultracentrifugation, using KBr solutions (Redgrave et al. 1975). Samples were centrifuged for 4 h at 4 °C at 190,000×*g* using a vertical rotor (Sorvall TV-860; Fisher Scientific) in a Sorvall WX Ultra Series Ultracentrifuge (Thermo Scientific). LDL fraction in KBr (1.019–1.063 g/ml) was recovered and desalted by gel filtration in PBS (pH 8) on a Sephadex G25 column (PD Miditrap G25; GE Healthcare). The protein content of LDL in PBS was measured by Folin protein determination assay (Lowry et al. 1951). Next, LDL was fluorescent-labeled with OG (Oregon Green^®^ 488 Carboxylic Acid, Succinimidyl Ester *6-Isomer*; Invitrogen). From this step, care was taken at any time to avoid exposure of OG(-LDL) to light. OG was dissolved in dimethylsulfoxide (20 μg/50 μl) and added to 1 ml of LDL (1 mg/ml), immediately vortexed and incubated for 45 min on an orbital shaker at 600 RPM, at RT. Unbound label was inactivated by adding 100 μl of glycine (0.01 M, final concentration) to the mixture and incubating under shaking for 15 min. LDL bound to OG (OG-LDL) was recovered by gel filtration in non-supplemented endothelial medium (EBM-2; Lonza), buffered with HEPES (pH 7.2; 25 mM final concentration; Invitrogen). Final protein concentration was determined by Folin assay.

### OG-LDL internalization by HUVECS

Seven-days cobblestone HUVECs were washed with warm EBM-2-HEPES and incubated with OG-LDL diluted in EBM-2-HEPES (250 μg protein/ml) for 1, 15 or 30 min at 37 °C (in a stove). After incubation cells were thoroughly washed to remove unbound/non-internalized OG-LDL and fixed with 4 % formaldehyde in 0.2 M HEPES for at least 30 min (max. 60 min) at RT. Fixative was washed out with PBS and cells stained with DAPI in PBS. Following washing, cells (on Aclar) were mounted with Prolong Gold as explained above. After curing, samples were analyzed by confocal laser scanning microscopy (LSM 5 Pascal; Carl Zeiss B.V.) as previously reported (Jiménez et al. 2010).

### Electron microscopy of HUVECs

In order to perform high-resolution analysis of HUVECs by transmission electron microscopy (TEM) and scanning electron microscopy (SEM), 7-days cobblestone cells were processed following protocols optimized to obtain an excellent cellular contrast. To this purpose, cells grown on Aclar were chemically fixed with aldehydes, post-fixed with OsO_4_, and osmium-impregnated by tannic acid exactly as reported (Jiménez et al. 2009). For TEM cells were then dehydrated and embedded in Epon (Jiménez et al. 2009). After polymerization, Aclar pieces were removed, Epon blocks trimmed and 60-nm thick sections cut either in parallel or perpendicular to the cell monolayer. Sections were collected on copper grids coated with Formvar and carbon. TEM analysis was performed in a Tecnai-12 microscope (FEI company) as previously described (Jiménez et al. 2006). For SEM cells were dehydrated in alcohol (Jiménez et al. 2009) and then passed to ethanol:acetone (1:1) mixture (2×) and pure acetone (2×). Aclar pieces, with HUVECs on, were then transferred to a critical point drier (CPD 030; Bal-Tec) and dried from carbon dioxide according the manufacturer’s manual. Pieces were attached to aluminium stubs using carbon tabs (Agar Scientific). Samples were sputtered with platinum/palladium to a thickness of 7 nm in a 208HR sputter coater (Cressington Scientific). SEM imaging (using secondary electrons) was done with a XL30-FEG microscope (FEI company) operating at an acceleration voltage of 5 kV and at a working distance of ~6 nm.

### Immuno-fluorescence and electron microscopy of umbilical veins

In order to get references to judge our HUVEC cobblestone model, pieces of two different umbilical cords were reserved to perform light and electron microscopy studies. The umbilical vein was cannulated, gently washed with HBSS and immediately perfused with fixatives. After a first fixation by perfusion, cords were cut with a scalpel in 0.5-cm long pieces that were immersed in fresh fixative.

For immuno-fluorescence analysis the vein was fixed with 1 % formaldehyde in HEPES for 45 min in total (15 min perfusion + 30 min immersion) at RT. After fixation, pieces were cut longitudinally to expose the endothelium of the vein. A layer of tissue, containing the *intima* and (at least part of) the *media* could then be easily pulled off from the umbilical cord using fine forceps. The manipulation of the umbilical tissue was aided by a stereomicroscope. Tissues were transferred to a 12-well plate, with endothelium upwards in the well, always taking care to prevent drying. Next, tissues were washed with PBS, blocked, quenched, permeabilized, and incubated with antibodies and DAPI as explained for HUVECs grown on Aclar. A droplet of Prolong gold was applied on a coverslip and the umbilical vein, with endothelium facing coverslip, was mounted on and left to cure overnight. Next day, samples were analyzed by confocal laser scanning microscopy (Jiménez et al. 2010).

For electron microscopy, the fixative was a mixture of glutaraldehyde and formaldehyde (Jiménez et al. 2009). Fixation with perfused aldehydes was done for 30 min at RT. The subsequent fixation by immersion, for at least 1 day at 4 °C. After washing out the aldehydes, the endothelium of the vein was exposed as just explained and pieces of approx. 2 mm^3^ were osmicated (Jiménez et al. 2009). After dehydration, samples were either embedded in Epon and sectioned for TEM analysis or critical point dried, mounted on aluminium stubs and sputtered with platinum/palladium as explained for HUVECs for SEM. A difference was that tissues were mounted on stubs using conductive carbon cement (Leit-C; Neubauer, Münster, Germany). Imaging with TEM and SEM was done as detailed for HUVECs.

## Results and discussion

### Sequential short trypsinization is a convenient and effective approach to harvest viable HUVECs

Trypsin(-EDTA) is a detachment enzyme commonly used in the cell culture practice. Trypsin (0.2 %) was used by Maruyama at the very first reported trial to isolate HUVECs from cannulated human umbilical veins (Maruyama 1963). Cells were harvested after 45 min of incubation at 37 °C. However, these cells in culture acquired variable morphologies and were not unequivocally characterized as HUVECs. Over-trypsinization is detrimental to cell viability as cells lose their capability to adhere to culture vessels (Anamelechi et al. 2009). Furthermore, it can also result in detachment of other cells underlying the endothelium. Not surprisingly, scientific community felt that the cells isolated by Maruyama were mixed cultures, and mainly fibroblasts (Nachman and Jaffe 2004). Some years later, Jaffe et al. published the first basic technique to establish *bona fide* cultures of HUVECs (Jaffe et al. 1973b). This method has been literally followed to culture HUVECs in vitro during the last 4 decades in thousands of studies. In recent years, technical publications appeared reporting protocols with modifications to the method (Baudin et al. 2007; Bazzoni et al. 2002; Davis et al. 2007; Laurens and van Hinsbergh 2004; Marin et al. 2001). In all of them, as in Jaffe’s paper, collagenase was the enzyme of choice to detach cells from the umbilical vein, and incubations happened at 37 °C (in a water bath). Collagenases carry diverse contaminating proteases and the optimal work concentration and incubation time is dependent on the specific type and batch of collagenase used to detach cells (Baudin et al. 2007).

Since trypsin is an ordinary enzyme with a highly reproducible activity, also at RT, we sought for isolating viable HUVECs by trypsinization following a protocol less demanding than the current ones. We found this by sequential short trypsinizations performed at RT, inside the laminar flow cast. Sequential incubations (5×, 2 min each) with injected trypsin–EDTA (0.05–0.022 %, respectively), combined with umbilical cord squeezing and intraluminal pressure build up and release, were enough to isolate sufficient viable vein endothelial cells to start up in vitro primary cultures (Online Resource 1). The trypsin used for one umbilical vein had been warmed up to 37 °C before the first injection and, inside the flow cast, cooled gradually down to approx. RT during the 10-min lasting procedure. Cell suspensions were kept at RT until the last eluate was recovered. Soon after the last cell recovery trypsin was neutralized, and therefore cells were exposed not longer than 10 min to the enzymatic activity. This procedure turned out to be mild while effective. All the umbilical cords used (n = 10) rendered cells able to attach to culture vessels coated with a thin layer of basement membrane matrix (Online Resource 1; “4 h after isolation, Matrigel”). Upon spreading cells acquired the long polygonal shape which characterizes non-confluent HUVECs in primary culture (Gimbrone et al. 1974; Jaffe et al. 1973b) (Online Resource 1; “40 h after isolation, Matrigel”).

The diameter of the lumen of at term umbilical veins varies from 3.1 mm at the proximal (placental) end to 2.3 mm at the distal (fetal) end (Li et al. 2006). Assuming an average diameter of 2.7 mm and a straight course of the vein, a 10-cm long segment of umbilical cord contains ~8.5 cm^2^ of endothelial vein surface. An umbilical vein endothelial cell in vivo has an approximate “diameter” of 15 μm (Online Resource 3; panels b and c) and covers a surface of ~180 μm^2^. According to this, a 10-cm long cord can yield about 4.5 × 10^6^ cells. Jaffe and others reported variable isolation efficiencies, from 0.3 × 10^6^ to 1.5 × 10^6^ cells for 20–30-cm long cords (Baudin et al. 2007; Jaffe et al. 1973b); hence, referred to a 10-cm long cord, the efficiency of isolation ranged from 3 to 11 % at best. After re-suspending harvested, pelleted cells in growth medium we tried to count them with a hemocytometer. Phase contrast microscopy showed single and clustered cells, as well as traces of blood cells. This made counting of the HUVECs unreliable. Therefore, we looked for an empirical rule to seed HUVECs. Based on the calculated endothelial vein surface and on reported isolation efficiencies, we judged that all the endothelial cells from a 10-cm long cord should fit on a 10-cm^2^ growth surface. Bearing this in mind, we seeded isolated cells in culture vessels following a “1:1, umbilical cord length:culture vessel surface” rule. Four h after isolation, cell medium was refreshed. In all cases (n = 10) we found attached cells (Online Resource 1; “4 h after isolation, Matrigel”). Cells were spreading and covered ~30 % of the surface (20–40 %). The “diameter” of spread cells was estimated to be, on average, 30 μm (i.e. a cell covers a surface of ~700 μm^2^). This means that, for our method, the efficiency of isolation of initially viable HUVECs (i.e. with ability to attach to culture vessels) ranges from 5 to 10 %, and therefore it is comparable to that from current protocols. Prolonged sequential trypsinization (more times, or longer incubations) increased cell harvest, but it is discouraged since it was paired to a high risk of culture contamination with long spindle-shaped cells (probably smooth muscle cells; data not shown).

The 2–3 days following isolation were crucial for the primary cultures. The lag phase during which cells “recovered” varied among isolations. In most cases, as soon as 20 h after harvesting, cells were widely spread and actively dividing. In 2 cases it seemed that cell growth stopped and cells were discarded. The other 8 cases progressed to cell densities of about 80 % confluency (70–90 %). As stated above, these cells showed a characteristic long polygonal shape (Online Resource 1; “40 h after isolation, Matrigel”) and were considered to be viable HUVECs.

### The differentiation of HUVECs into a mature cobblestone monolayer can be assessed by ordinary phase contrast microscopy

Viable primary cultures (n = 8) were used to obtain cobblestone monolayers on Matrigel, in all cases with success. Cells were seeded at 20,000 cells/cm^2^ and monitored by phase contrast microscopy at different stages. In subconfluent cultures mitotic figures were easily found, and HUVECs were very spread (Fig. 1; “subconfluent”). After 4 days the monolayer reached 100 % confluence and cells, remarkably smaller, started to show a cobblestone appearance. In no case we observed contamination with smooth muscle-like or fibroblast-like cells. The first days, the limits of single cells appeared bright under phase contrast (Fig. 1; “2-days cobblestone”). This changed gradually and after a couple of days, cell limits begun to appear dark while cell organelles seemed to accumulate around the nuclei (Fig. 1; “4-days cobblestone”). Some days later, the dark cellular limits became clearly patent and organelles seemed to be highly concentrated in the peri-nuclear area (Fig. 1; “7-days cobblestone”). This latter phenotype of the cell–cell contact area resembled a mature, tight-cobblestone state (Smeets et al. 1992), which was confirmed by further characterization (see below). Therefore, simple monitoring of HUVECs by phase contrast microscopy can indicate when the cobblestone monolayer has reached the mature state. Prolonged culture periods did not affect the aspect of the cobblestone monolayer but were associated to appearance of “sprout” cells that acted as overgrowth foci. Overgrowth in long-term HUVECs cultures is a known phenomenon (Smeets et al. 1992) that should be avoided.

### The 7-days cobblestone HUVECs model shows important characteristics of the human umbilical vein endothelium in vivo

The continuous endothelium of the blood vessels is a monolayer where cells show a cobblestone appearance. One of the most important functions of the endothelium is to separate blood from underlying tissues and to act as selective filter for water, solutes and cells. Cell–cell junctions (tight junctions and adherens junctions) are responsible for the maintenance of the integrity of the endothelium (Dejana 2004) and therefore pivotal for a functional barrier. The selective transport of plasma proteins into the subendothelial space is mainly mediated by caveolae, plasma membrane invaginations coated with caveolins (Lebbink et al. 2010). In addition, endothelial cells are also involved in hemostasis since they produce and secrete von Willebrand factor (vWF) (Wagner et al. 1982), which is stored in the endothelial-specific Weibel-Palade bodies (Weibel and Palade 1964).

**Fig. 2:**
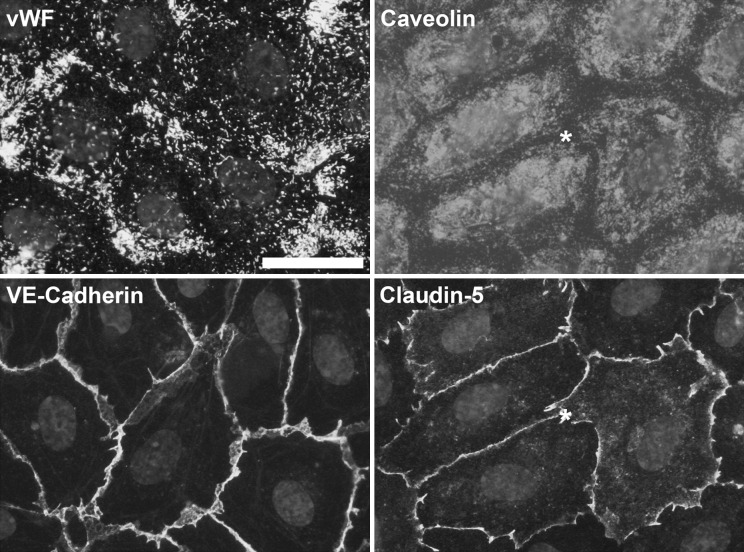
Characterization of 7-days cobblestone HUVECs by immuno-fluorescence. Cells (passage 1) were seeded at 20,000 cells/cm^2^ on Aclar coated with Matrigel. When cells reached the 7-days cobblestone state, they were fixed and immuno-labeled. Nuclei were counterstained with DAPI. Cells express endothelial markers. vWF is found in discrete cytoplasmic structures and caveolin is present from the nuclear to the cell–cell contact area. Labeling for VE-cadherin (component of the adherens junctions) and claudin-5 (part of the tight junctions) is well defined at the cell periphery as expected for a tight cobblestone monolayer. *Asterisks* mark the same cell. *Scale bar* (applicable to all the *panels*): 30 μm

**Fig. 3:**
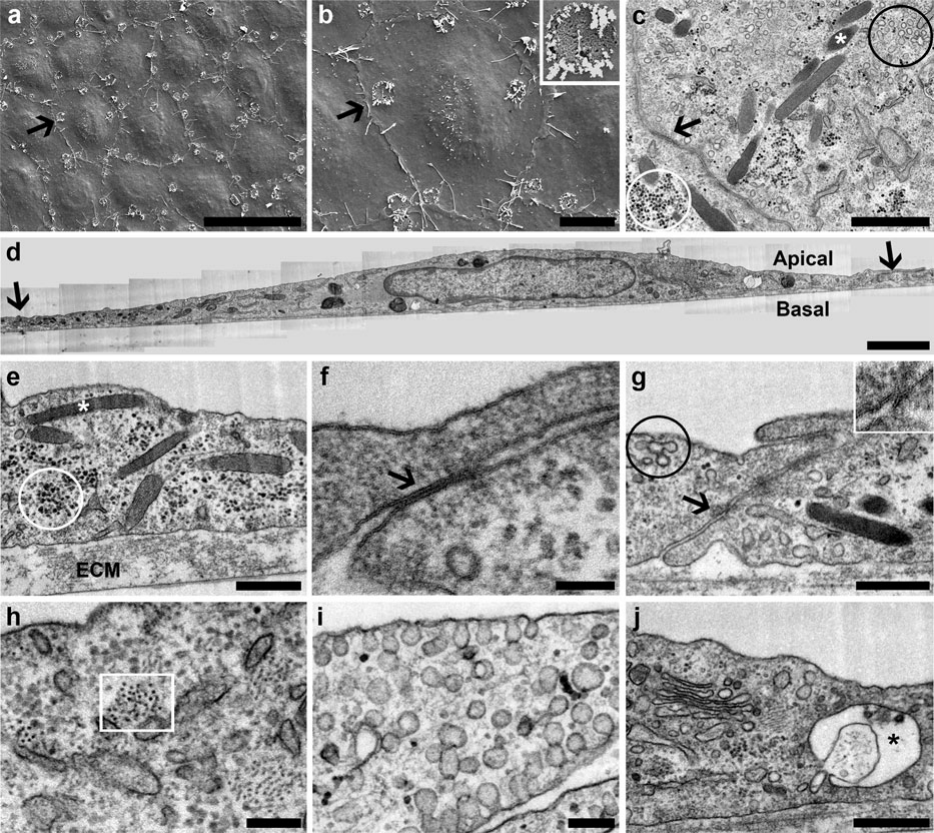
Characterization of 7-days cobblestone HUVECs by electron microscopy. HUVECs (passage 1) were seeded at 20,000 cells/cm^2^ on Aclar coated with Matrigel. Upon reaching the 7-days cobblestone state, cells were fixed and processed for SEM or TEM. **a**, **b** Scanning electron microscopy reveals a tight cell monolayer with well-defined cell limits. Pores (detail at inset in b) are found in the plasma membrane. *Arrows point* the same cell in the cobblestone. **c**–**j** Transmission electron microscopy of thin sections cut either in parallel (**c**) or perpendicular (**d**–**j**) to the cell monolayer shows diverse features associated to the umbilical vein endothelium in vivo; namely: Weibel-Palade bodies (**c**, **e**, *white asterisks*), caveolae (**c**, **g**, *black circle*; **i**), adherens junctions (**f**, *arrow*), tight junctions (**g**, *arrow* and inset), intermediate filaments (**h**, *white square*), Golgi complex (**j**), and glycogen inclusions (**c**, **e**, *white circle*). Structures resembling secretory pods, recently described for HUVECs in culture (Valentijn et al. 2010), are also present (**j**, *black asterisk*). HUVECs lie on a well-developed extracellular matrix (**e**, ECM). In d, a panoramic of a complete cell is shown; therein, as in c, *arrows point* cell limits. *Scale bars*: **a** 50 μm; **b** 10 μm; **c** 1 μm; **d** 2 μm; **e**, **g**, **j** 500 nm; **f** 100 nm; **h**, **i** 200 nm

We characterized our 7-days cobblestone HUVECs model (established on Matrigel) using different approaches. Cells seeded on Aclar formed a 7-days cobblestone monolayer (Online Resource 2; “Matrigel”) similar to the one obtained on ordinary culture plastic (Fig. 1; “7-days cobblestone”). Then, the expression of vWF, caveolin and junctional proteins (VE-cadherin as component of adherens junctions and claudin-5 as component of tight junctions (Dejana 2004)) was studied by immuno-fluorescence. vWF was found in all the cells as punctate structures, frequently clustered (Fig. 2). Caveolin was very abundant and present uniformly from the nuclear region to near the junctional area (Fig. 2). VE-cadherin and claudin-5 were localized at the cell–cell contact areas, and formed a continuous band following the cell limits (Fig. 2). In negative controls, where the primary antibody had been omitted, no fluorescent signal was detected (data not shown). Once low resolution microscopy showed that 7-days cobblestone HUVECs express important endothelial markers we switched to high resolution studies for a better unequivocal characterization of our model. Transmission electron microscopy (Fig. 3 c–j) gave clear evidences of the presence of the endothelial specific Weibel-Palade bodies (Fig. 3c, e, white asterisks), well-formed tight junctions (Fig. 3g, arrow and inset) and adherens junctions (Fig. 3f, arrow) and multitude of caveolae with diverse morphological manifestations (Fig. 3c, g, circled in black; Fig. 3i). We observed other ultrastructural details described for HUVECs in culture (Elgjo et al. 1975; Jaffe et al. 1973a) as a prominent Golgi (Fig. 3j) and intermediate filaments (Fig. 3h, squared in white). As expected, all the referred features were also observed by TEM in endothelial cells of the umbilical vein in situ (Online Resource 3; see figure legend for explanation). Inclusions of glycogen were another characteristic of the endothelium in vivo (Online Resource 3; panel i, circled in white) that was observed in cultured HUVECs (Fig. 3c, e, circled in white). We also found, in vivo and in vitro, structures resembling the recently described secretory pods (Valentijn et al. 2010) (Fig. 3j and Online Resource 3, panel i; black asterisks). The *intima* in vivo (i.e. the endothelium) was separated from the *media* (i.e. smooth muscle cells) by a prominent *elastica interna* (Online Resource 3; panel d). Similarly, HUVECs in culture reposed on a well-developed extracellular matrix (ECM; clearly visible in Fig. 3e). Based on previous reports this extracellular matrix might be formed by fibronectin, basement membrane collagens and laminin (Jaffe et al. 1976; Jaffe and Mosher 1978; Levene et al. 1988).

Scanning electron microscopy has revealed that intact endothelial cells of the umbilical vein are elongated in the direction of the blood flow (Akers et al. 1977). Fluid shear stress is responsible for the organization of the actin cytoskeleton and therefore for the endothelial cell shape (Franke et al. 1984). For our study, umbilical veins were collected after birth and kept at 4 °C for 24 h before fixation. Not surprisingly, scanning electron micrographs of the endothelium in vivo showed non-elongated polygonal cells (Online Resource 3; panels b and c) probably due to the cytoskeleton disassembly under static conditions. In the umbilical vein, endothelial cells were not tightly packed (Online Resource 3; panels b and c), which could be explained by the disorganization of the junctional complexes observed by confocal laser scanning microscopy (Online Resource 3; panel a). Interestingly, this loose disposition of cells in the monolayer can explain the rapid penetration of trypsin under the endothelium and thus the relatively fast cell detachment. SEM of 7-days cobblestone HUVECs revealed tightly packed polygonal cells, with a distinct cellular limit and a protruding nuclear area (Fig. 3a, b). These observations could be easily related to those from phase contrast microscopy (Online Resource 2; “Matrigel”). The plasma membrane was decorated with pores (Fig. 3b, inset) and showed several short microvilli (Fig. 3b). The pores are likely the opening of the secretory pods to the luminal space (Valentijn et al. 2010). Pores were also observed in the endothelium in vivo (Online Resource 3; panels b and c) and were similar to those found on the surface of the coronary artery endothelium (Reichlin et al. 2005). A difference between endothelial cells in situ and HUVECs in vitro was the cell size. As stated above, cells in the vein were small, with a “diameter” of ~15 μm (Online Resource 3; panel b). In contrast, cobblestone HUVECs in culture had a “diameter” of approx. 40 μm (Fig. 3a). Accordingly, TEM of cells sectioned perpendicularly to the monolayer showed a more extensive spreading of HUVECs (Fig. 3d) as compared to the endothelial cells in situ (Online Resource 3; panel d). This observation was not unexpected. The area occupied by a single HUVEC in culture increases with the population doubling times (Hasegawa et al. 1988; Kiyonaga et al. 2001) and our HUVECs had proliferated before forming the cobblestone.

Following the low and high resolution characterization we performed functional assays with the 7-days cobblestone HUVECs model. A well-established function of endothelium in vivo is the transcellular transport of lipoproteins (Vasile et al. 1983). Seven-days cobblestone HUVECs were incubated with low-density lipoprotein tagged with fluorescent Oregon Green (OG-LDL) for different times (1, 15 or 30 min). After thorough washing, cells were fixed and processed for visualization with confocal laser scanning microscope. As soon as 1 min after addition, cells had already internalized OG-LDL, that appeared in punctate structures in the cytoplasm (Online Resource 4; “1′ OG-LDL”). These distinct structures disappeared gradually concomitant with the appearance of a diffuse labeling (Online Resource 4; “15′ OG-LDL”, “30′ OG-LDL”). The diffuse labeling was visible in the most basal optical slice of the stacks. Our results indicate that the 7-days cobblestone HUVECs are able to internalize LDL that can move across the cells via transcytosis. The exact mechanism of transport remains to be elucidated.

### The 7-days cobblestone HUVECs model can be established on different extracellular matrices

Endothelial cells in vivo lie on a basal lamina. Laminin, collagen type IV, entactins and heparan sulfate, important constituents of the lamina, are also the main components of soluble extracts of basement membrane (Kleinman et al. 1982), commercially available as, for example, Matrigel (BD Biosciences) or Geltrex (Invitrogen). Applied as thick, gelled coating, Matrigel acts as 3D matrix in which endothelial cells form capillary-like structures (Lawley and Kubota 1989). Used as thin, non-gelled coating, Matrigel supports the formation of an endothelial cell monolayer (Martins-Green et al. 2008). By virtue of its composition, we judged Matrigel (applied as thin coating) to be a good starting point to set up endothelial cobblestone cultures. Indeed, the results presented so far in this paper have shown that HUVECs can be isolated and properly differentiated on Matrigel.

Adhesion to Matrigel is probably mediated by receptors, expressed by HUVECs, which bind laminin and collagen (Albelda et al. 1989). However, HUVECs also express receptors for collagens and fibronectin (Albelda et al. 1989). Not surprisingly, fibronectin and gelatin (i.e. a mixture of collagen derivatives) have been routinely used as coating to grow HUVECs on (Baudin et al. 2007; Laurens and van Hinsbergh 2004; Marin et al. 2001). We aimed to compare the isolation and differentiation of HUVECs (from n = 2 different cords) in parallel on Matrigel, fibronectin and gelatin. In all cases, coating solutions were applied following the same protocol and left to coat by adsorption. All coatings allowed for establishment of primary HUVECs. Soon after isolation attached cells were found on all matrices, although cells seemed to be more spread on fibronectin (Online Resource 1; “4 h after isolation”). About 2 days later, cells had acquired a similar elongated-polygonal morphology on all substrates (Online Resource 1; “40 h after isolation”). These results are in agreement with previous observations on primary HUVECs seeded on different coatings (Macarak and Howard 1983). Passage 0 HUVECs were sub-cultured (20,000 cells/cm^2^) on Aclar coated with the corresponding matrix and left to differentiate into a cobblestone morphology. In all cases cells reached confluence and formed a cobblestone monolayer following a similar timing. HUVECs formed a mature 7-days cobblestone monolayer not only on Matrigel, but also on fibronectin and gelatin, as judged by phase contrast microscopy (Online Resource 2). As detailed for Matrigel, immuno-fluorescence for vWF, caveolin, VE-cadherin and claudin-5, as well as electron microscopy validated 7-days cobblestones on fibronectin and gelatin as endothelium-mimicking monolayers (data not shown). It is, however, important to mention that cultures on gelatin showed “sprout” cells already in the early cobblestone. These cells triggered an extensive overgrowth in 7-days cobblestone cultures. Therefore, although valid, mature cobblestone cells can be established on basement membrane, fibronectin and gelatin matrices, in our view Matrigel and fibronectin offer a higher chance to get a successful culture. Since fibronectin is relatively expensive, Matrigel might become a very good alternative for culture of HUVECs.

## Conclusions

In the present study we have shown that: (1) viable endothelial cells from human umbilical veins can be easily and efficiently harvested by sequential short trypsinization; (2) HUVECs, isolated following our protocol and cultured for 7-days as cobblestone on basement membrane matrix, fibronectin or gelatin, form a mature tight cell monolayer that mimics the human umbilical vein endothelium in vivo; and (3) the morphology of the 7-days cobblestone HUVECs model is so characteristic that can be used as criterion to determine, by ordinary phase contrast microscopy, when a cobblestone monolayer becomes mature.

## Electronic supplementary material

Below is the link to the electronic supplementary material.

Supplementary material 1 (PDF 236 kb)

Supplementary material 2 (PDF 127 kb)

Supplementary material 3 (PDF 371 kb)

Supplementary material 4 (PDF 125 kb)
