# Supplementary material for: A new, rapid and reproducible method to obtain high quality endothelium in vitro
Source: Cytotechnology. 2012 May 10;65(1):1–14. doi: 10.1007/s10616-012-9459-9 (PMC3536875; doi:10.1007/s10616-012-9459-9)
Supplement: Supplementary file 1 — Supplementary material 1 (PDF 236 kb) [file 10616_2012_9459_MOESM1_ESM.pdf]

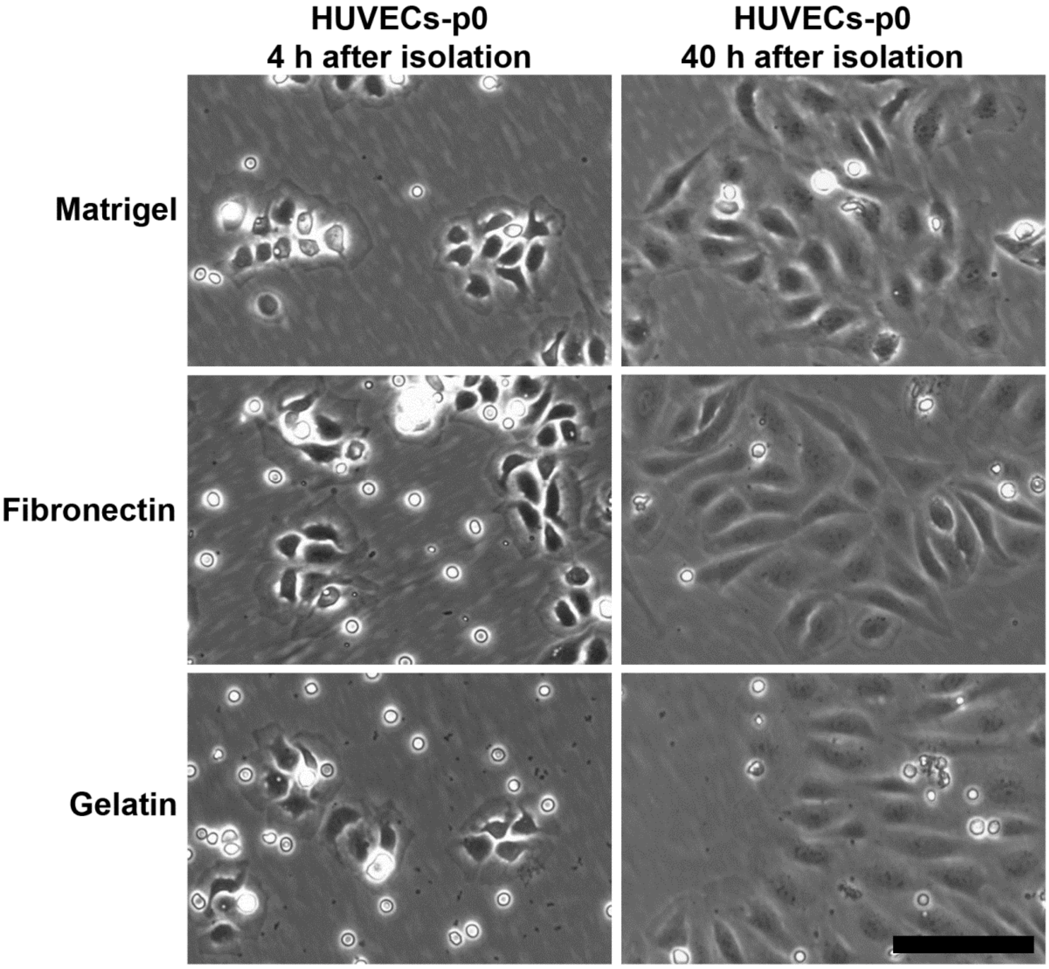

Online Resource 1 ***Isolation of HUVECs on Matrigel, fibronectin and gelatin.*** Cells were harvested by sequential short trypsinization and seeded on coated vessels (passage 0, p0). Four hours after isolation, cells have attached to all substrates and are already spreading. Forty hours after seeding, cells are extensively spread and show characteristic elongated-polygonal shape on every coating. Scale bar (applicable to all the panels): 100 µm
