# Supplementary material for: A new, rapid and reproducible method to obtain high quality endothelium in vitro
Source: Cytotechnology. 2012 May 10;65(1):1–14. doi: 10.1007/s10616-012-9459-9 (PMC3536875; doi:10.1007/s10616-012-9459-9)
Supplement: Supplementary file 2 — Supplementary material 2 (PDF 127 kb) [file 10616_2012_9459_MOESM2_ESM.pdf]

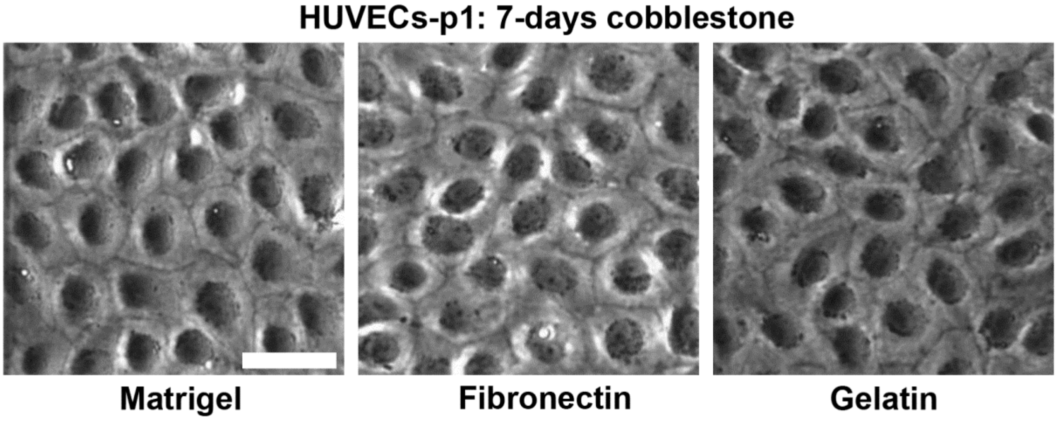

Online Resource 2 ***Phase contrast microscopy of 7-days cobblestone HUVECs on Matrigel, fibronectin and gelatin.*** Cells (passage 1, p1) were seeded at 20000 cells/cm^2 on Aclar coated with different matrices and left to reach the 7-days cobblestone state. In all the cases, cells show distinct dark cell limits associated to the mature cobblestone appearance. Scale bar (applicable to all the panels): 50 µm
