# Supplementary material for: A new, rapid and reproducible method to obtain high quality endothelium in vitro
Source: Cytotechnology. 2012 May 10;65(1):1–14. doi: 10.1007/s10616-012-9459-9 (PMC3536875; doi:10.1007/s10616-012-9459-9)
Supplement: Supplementary file 3 — Supplementary material 3 (PDF 371 kb) [file 10616_2012_9459_MOESM3_ESM.pdf]

A new, rapid and reproducible method to obtain high quality endothelium *in vitro*
Cytotechnology
Nuria Jiménez, Vincent J.D. Krouwer and Jan A. Post

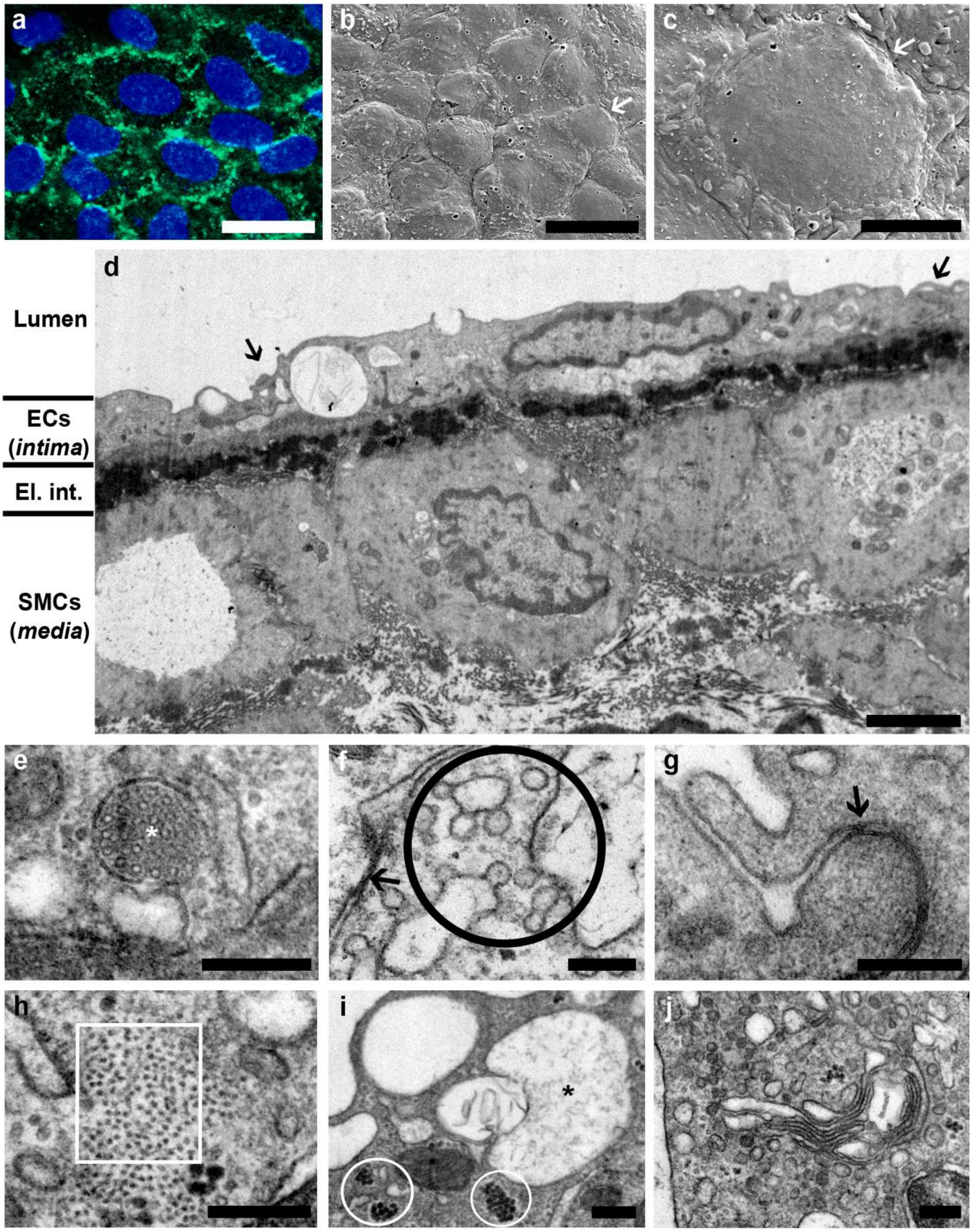

Online Resource 3 ***Characterization of the umbilical vein endothelium in situ.*** Umbilical veins were fixed by perfusion and immersion 24 h after birth and processed for immuno-fluorescence or for electron microscopy. (**a**) Confocal laser scanning microscopy of umbilical vein endothelium labeled for VE-cadherin. The junctional area is disorganized after 24 h of cord storage at 4 °C under static conditions. (**b, c**) Scanning electron microscopy show small polygonal cells that seem to retract. Cells have pores on the plasma membrane. Arrows point out the same cell. (**d-j**) Transmission electron microscopy of sections cut perpendicularly to the endothelium. A panoramic view of the *intima* (with endothelial cells, ECs), *elastica interna* (El. int.) and part of the *media* (with smooth muscle cells, SMCs) can be found in d; arrows point the limits of one endothelial cell. Diverse endothelial features, that can also be found in 7-days cobblestone HUVECs, are detailed; namely: Weibel-Palade bodies (e, white asterisks), caveolae (f, black circles), adherens junctions (f, arrow), tight junctions (g, arrow), intermediate filaments(h, white square), Golgi complex (j), and glycogen inclusions (i, white circles). Structures resembling secretory pods are also present (i, black asterisk), just as in HUVECs. Scale bars: a, b, 20 µm; c, 5 µm; d, 2 µm; e-j, 200 nm
