# Supplementary material for: A new, rapid and reproducible method to obtain high quality endothelium in vitro
Source: Cytotechnology. 2012 May 10;65(1):1–14. doi: 10.1007/s10616-012-9459-9 (PMC3536875; doi:10.1007/s10616-012-9459-9)
Supplement: Supplementary file 4 — Supplementary material 4 (PDF 125 kb) [file 10616_2012_9459_MOESM4_ESM.pdf]

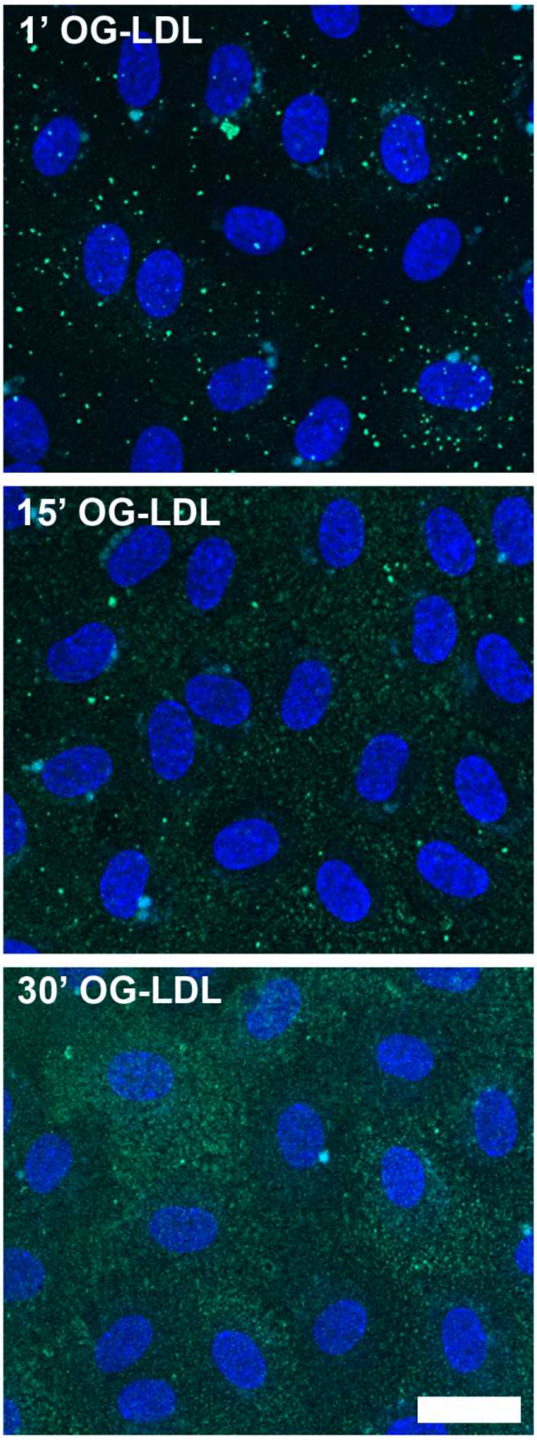

Online Resource 4 ***OG-LDL uptake by 7-days cobblestone HUVECs.*** When HUVECs (passage 1) reached the 7-days cobblestone state, they were incubated with OG-LDL for 1, 15 or 30 min. After incubation cells were thoroughly washed, fixed, counterstained with DAPI and mounted with Prolong Gold. Stacks of optical slices (0.44 µm thick) were taken by confocal laser scanning microscopy to image the whole cells. The pictures shown correspond to the most basal optical slice. HUVECs internalize OG-LDL very fast. After 1 min of incubation, OG-LDL is found in discrete points in the cytoplasm. As the incubation time progresses, a diffuse basal labeling appears and increases while the distinct OG-LDL positive structures decrease. Scale bar (applicable to all the panels): 20 µm
